# Multiple pigmented squamous cell carcinoma *in situ* on the volar hands after chronic use of topical tacrolimus

**DOI:** 10.1016/j.jdcr.2024.07.012

**Published:** 2024-08-03

**Authors:** Hailey Konisky, Alison Kortz, Albert Huho, A. Neal Gregory

**Affiliations:** a Albert Einstein College of Medicine, Bronx, New York; b Upstate Dermatology, Castleton-on-Hudson, New York

**Keywords:** Bowen’s disease, immunomodulator, squamous cell carcinoma in situ, tacrolimus

## Introduction

Bowen’s disease (squamous cell carcinoma *in situ*) is a condition that typically presents as scaly erythematous lesions on sun-exposed areas, most often in Caucasians over the age of 60.^1^ In less than 2% of cases, these lesions are pigmented. Pigmented Bowen’s disease is most common in Black men; however, to our knowledge, there are only 2 other cases reported on volar surfaces.^1^, ^2^, ^3^ Herein, we describe a novel case of multiple pigmented Bowen’s disease lesions on the volar hands of a man after chronic topical tacrolimus use.

## Report of a case

**Fig 1:**
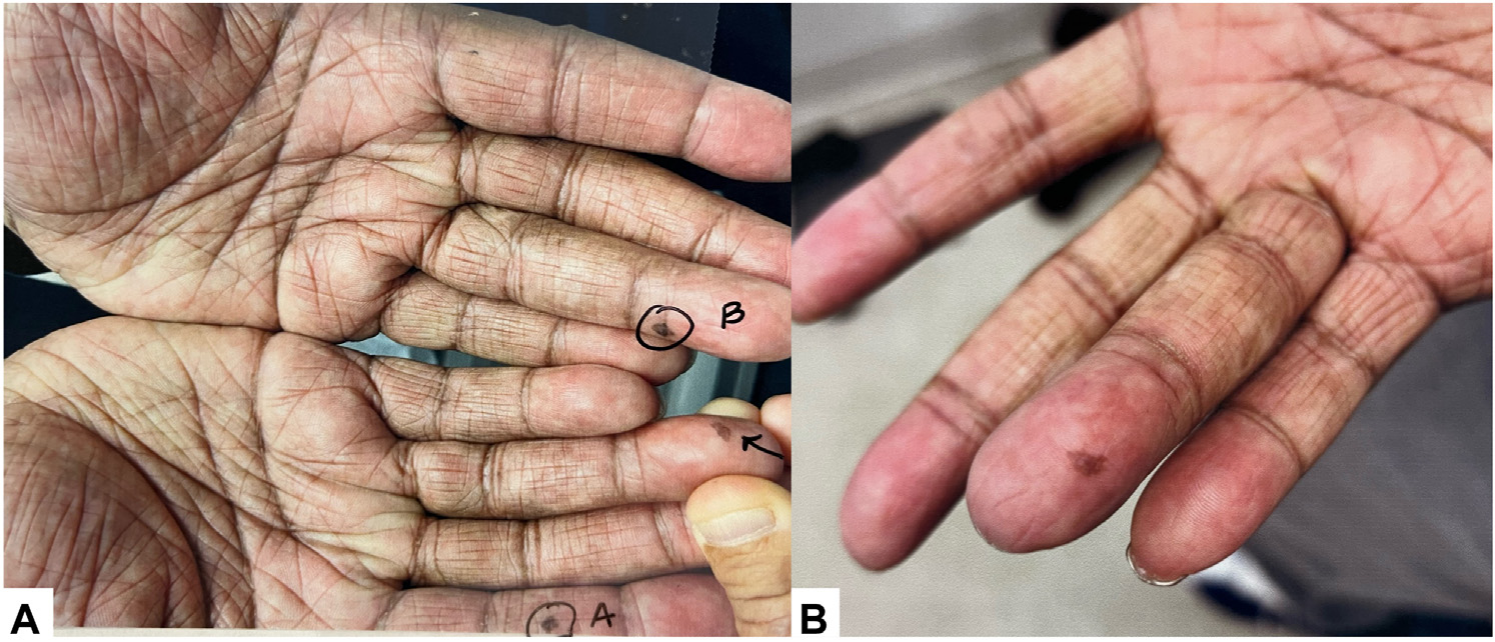
**A,** Photo of all 3 lesions (A, B, *arrow*) prior to biopsy. **B,** Zoomed in view of lesion on the right distal fourth digit prior to biopsy.

**Fig 2:**
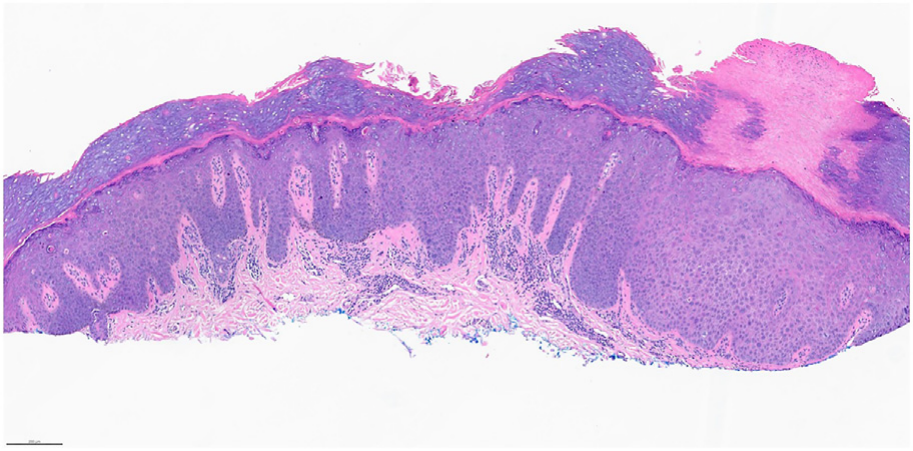
Pathology of one of the pigmented macules showing atypical keratinocytes present throughout the entire thickness of a pigmented epidermis.

A 55-year-old man with Fitzpatrick Skin Type V presented with 3 irregular pigmented macules on his right distal volar first and fourth digits and his left distal volar fourth digit for less than 6 months, concerning for acral melanoma (Fig 1). He had a long-standing history of asthma and chronic lichenified atopic dermatitis (AD) affecting over 90% of body surface area, with improvement after 4 years of daily topical tacrolimus 0.1% to the hands and 3 years of twice-monthly dupilumab 300 mg. The patient previously failed trials of narrow-band ultraviolet B therapy. Dermoscopy showed irregular streaks of pigment not following either a ridge or furrow pattern. Punch biopsies of all 3 lesions noted atypical keratinocytes present throughout the entire thickness of a pigmented epidermis, consistent with squamous cell carcinoma *in situ,* or Bowen’s disease (Fig 2). The patient denied known exposure to arsenic, human papilloma virus, or extensive sun exposure. Biopsies were all negative for human papilloma virus 16 and 18 by *in situ* hybridization, and he had no history or evidence of verruca clinically. Treatment options (including 5-fluorouracil, cryotherapy, photodynamic therapy, and surgical excision) and risk of recurrence were discussed with the patient and he opted for a 6-week course of imiquimod.

## Discussion

Pigmented Bowen’s disease of the volar hands is an exceedingly rare condition with risk factors including exposure to sunlight, chronic arsenic exposure, radiotherapy, Human papilloma virus infection, and trauma.^3^ In the case of our patient, he had an extensive history of AD on his hands treated with topical tacrolimus and remote history of ultraviolet B therapy. Given the patient’s skin type and the lack of squamous cell carcinoma (SCC) elsewhere on his skin, we do not think the ultraviolet B therapy is implicated. Furthermore, the tacrolimus was exclusively used on his hands as his AD was not responsive to dupilumab in this area. Generally, SCC is the most common skin cancer in skin of color patients, often arising in areas of chronic inflammation.^4^ Tacrolimus is an Food & Drug Administration-approved calcineurin inhibitor that locally suppresses the immune system to treat AD. It has been associated with an increased risk of SCC in some reports. Two case reports noted SCC at the site of topical tacrolimus use: one in a patient with oral lichen planus and the second in a man with balanoposthitis. The authors hypothesized that tacrolimus may have allowed for the growth of pre-existing malignant cells, which may have been present in our patient after years of inflammation on his volar hands.^5^^,^^6^

To date, there have been no other published cases of multiple Bowen’s disease lesions associated with chronic topical tacrolimus use. This case highlights the importance of considering this diagnosis in patients with new pigmented lesions in areas of chronic topical tacrolimus use, especially in patients with skin of color.

## Conflicts of interest

None disclosed.
